# A Network Analysis of Depressive Symptoms in the Elderly with Subjective Memory Complaints

**DOI:** 10.3390/jpm12050821

**Published:** 2022-05-18

**Authors:** Sunhae Kim, Kounseok Lee

**Affiliations:** Department of Psychiatry, Hanyang University Medical Center, Seoul 04763, Korea; sunhk0906@hanyang.ac.kr

**Keywords:** subjective memory complaints, depression, network analysis, symptoms, cognitive decline, depressive mood

## Abstract

(1) Background: Subjective memory complaints (SMCs) are common among the elderly and are important because they can indicate early cognitive impairment. The factor with the greatest correlation with SMCs is depression. The purpose of this study is to examine depressive symptoms among elderly individuals with SMCs through a network analysis that can analyze disease models between symptoms; (2) Methods: A total of 3489 data collected from elderly individuals in the community were analyzed. The Subjective Memory Complaints Questionnaire and Patient Health Questionnaire-9 were evaluated. For statistical analysis, we investigated the features of the depressive symptoms network, including centrality and clustering; (3) Results: Network analysis of the SMC group showed strong associations in the order of Q1–Q2 (r = 0.499), Q7–Q8 (r = 0.330), and Q1–Q6 (r = 0.239). In terms of centrality index, Q2 was highest in strength and expected influence, followed by Q1 in all of betweenness, strength, and expected influence; (4) Conclusions: The network analysis confirmed that the most important factors in the subjective cognitive decline group were depressed mood and anhedonia, which also had a strong correlation in the network pattern.

## 1. Introduction

### 1.1. Subjective Memory Complaints

Subjective memory complaints (SMCs) are a frequent phenomenon in the elderly population [1,2,3]. Discomfort regarding memory loss is common in everyday life, and this phenomenon is generally thought to be related to aging. This has been supported by many studies comparing memory in various age groups with normal controls by measuring and comparing various aspects of memory in the elderly in the secondary memory process to young people [4].

People with SMCs may be at an increased risk of dementia and have a higher rate of progression to mild cognitive impairment (MCI) or Alzheimer’s disease (AD) than patients without SMCs [5,6,7,8,9], suggesting that SMCs represent the “evolution of normal aging into clinical AD.” Individuals with SMCs may be in the pre-MCI stage and be a potential target for intervention trials. However, the clinical significance of SMCs [10], whether additional evaluation is necessary, and their differentiating characteristics remain unclear.

In addition, SMCs frequently appear in epidemiological surveys of 40–80% [1,5] of the community compared with 18.6–26.1% of those seeking clinical help [5,11,12]. Although the prevalence of SMCs is low compared with the prevalence of seeking therapeutic help or being diagnosed in primary care, there is increasing evidence that it may be related to the risk of dementia [13,14]; therefore, screening and intervention are important issues.

Forgetfulness is a delay or slowness in remembering something. It may be part of the normal aging process. In contrast, memory problems that interfere with daily life can be pathological. In the elderly, age-related memory changes are described as “benign senescent forget fullness (similarly, Mild memory defect) [15].” The National Institute of Mental Health has set out a study standard for this phenomenon, named age-associated memory impairment (AAMI) [16], which includes progressive memory loss in a person’s day-to-day problems. The presence or absence of discomfort is also included as an essential criterion of the AAMI concept; therefore, it is very important to know whether such discomfort is related to the results of a memory test or to other factors, such as personality traits or the subject’s emotional state. Many studies have reported a significant relationship between memory discomfort and memory performance assessed by memory tests [17,18,19,20]. However, other researchers have found no association or only weak associations between subjects’ performance on memory tests and memory discomfort questionnaires. In these studies, memory discomfort was associated with depressed mood [21,22,23,24,25] or anxiety or stress [26], poor social networks, negative stereotypes about aging, poor emotional state [27], indecisiveness, difficulty concentrating, mental deterioration [28], and neurotic tendencies [29]. As memory discomfort also reflects a tendency toward somatic dissatisfaction, it is often concluded that memory discomfort is more characteristic of a disease process than organic [30].

### 1.2. Depression and SMCs

The factor most strongly correlated with SMCs is depression rather than cognitive ability, and this finding has been consistently shown in previous studies [23,31,32,33,34,35]. There are few or inconsistent cross-sectional studies showing a strong relationship between SMCs and objective performance [28,36,37,38,39,40,41], suggesting that depressive symptoms are a major factor in SMCs.

Depressive symptoms are common in 10–15% of the elderly [42], and 8–20% experience severe depressive symptoms [43,44]. In addition, old-age depression has different characteristics from depression among young adults. Young adults mainly complain of guilt and sexual dysfunction, whereas, in the case of the elderly, symptoms such as decreased vocational ability, psychomotor retardation, and anxiety are prominent [45]. In addition, since most of the elderly experience discomfort due to physical symptoms, diagnostic discrimination of depressive disorder from the general aging process through common physical symptoms is difficult [46]. In general, cognitive impairment in depression is due to decreased hippocampal volume due to an impaired hypothalamus–pituitary adrenal axis, increased corticosteroid levels, and anterior cingulate gyrus dysfunction. Thus, neuropsychological assessments of depressed people show decreased psychomotor speed, attention, memory, and executive function compared with assessments of healthy controls [47]. In particular, this decline is more difficult to discriminate in the elderly.

As such, although depression is the most important predictor of SMCs, the present cognitive performance score (e.g., Mini-Mental State Examination (MMSE) score) is also significant [14], suggesting that subjective memory discomfort should not be considered secondary to depression [48]. Therefore, despite the primary importance of identifying depressive symptoms in patients with cognitive impairment, depression in elderly patients with cognitive impairment is different from general depression.

### 1.3. Psychiatric Research and Network Analysis

Network analysis is a research method that complements the limitations of the traditional statistical approach and considers that symptoms of psychopathology are expressed by common underlying factors [49]. In other words, just as patients with brain tumors suffer from headaches, depressive disorder acts as a base factor that causes symptoms such as depressed mood, decreased interest, weight changes, sleep disturbances, fatigue, and suicidal thoughts. This is called a disease model, and the disease model has shortcomings in explaining clinical features that vary according to the patient’s environment or condition. Accordingly, it is necessary to carefully examine the relationship between symptoms rather than to view mental disorders as sets of individual symptoms. Network analysis has strength in this regard. A network consists of nodes that represent constituent factors and edges that represent relationships between nodes. Through network analysis, the correlation between nodes not only can be checked but also has the advantage of being able to identify key nodes by determining which nodes are strongly associated with neighboring nodes [50].

When the symptoms of psychopathology are implemented as a network, a core node is also called a central symptom. When this core node is activated, other related symptoms are expressed, and the entire network is activated, which affects the onset and chronicity of psychopathology [49]. Although network analysis has some similarities to existing research methods, there are several differentiating features. Unlike the latent variable model, which assumes equal significance of symptoms of specific psychopathologies, network analysis has the advantage of being able to identify key symptoms that play important roles in the onset and maintenance of psychopathology [49].

### 1.4. Study Purpose

Subjective memory complaints predict decreased cognitive function in old age and can be used to screen for symptoms of more severe cognitive impairment. Many studies have revealed that depression causes greater neuropsychological damage in individuals with SMCs [28,51,52,53], and it is a highly correlated factor in the elderly. Therefore, in this study, the core symptoms of depression in elderly individuals with subjective cognitive discomfort were identified through the network analysis technique.

## 2. Materials and Methods

### 2.1. Participants

Data from 3489 subjects aged 60 and over surveyed in 2020 and 2021 in Yangpyeong, Gyeonggi-do, Korea, were used. Subjects agreed to participate in the study with confirmation of anonymity, and a questionnaire was conducted on those who agreed to this; if they did not agree, the survey was suspended. This study was approved by the Institutional Review Board of Hanyang University Hospital (HYUHIRB-2022-03-028).

### 2.2. Measurements

#### 2.2.1. Subjective Memory Complaints Questionnaire (SMCQ)

The Korean version of the Subjective Memory Decline Questionnaire (SMCQ) is a tool to evaluate the subjective memory loss MONF in the elderly [54]. Subjective memory loss is divided into three factors: general memory loss, everyday life memory loss, and subjective memory loss. The SMCQ consists of 14 items to evaluate subjective memory loss symptoms. Among them, four questions (Subjective Amnesia Questionnaire Questions 1–4) measure overall memory decline, and 10 (Subjective Amnesia Questionnaire Questions 5–14) measure daily life memory loss [54]. The cut-off point was 5/6, and 525 out of 3489 patients were classified as the SMC group.

#### 2.2.2. Patient Health Questionnaire-9 (PHQ-9)

The Patient Health Questionnaire-9 (PHQ-9) is a tool developed for the diagnosis of major depressive disorder (MDD) and was designed in accordance with the diagnostic criteria for major depressive episodes of the DSM-IV [55]. The PHQ-9 consists of nine items: Q1 (anhedonia), Q2 (depressed mood), Q3 (sleep problems), Q4 (low energy), Q5 (appetite change), Q6 (low self-esteem), Q7 (concentration difficulties), Q8 (psychomotor agitation or retardation), and Q9 (suicidal ideation) in the past 2 weeks. The score of each item ranges from 0 to 3 points, and the total score ranges from 0 to 27 points. A higher score indicates more severe depressive symptoms. The Korean version of the PHQ-9, the reliability and validity of which have been demonstrated in standardization studies in Korea, was used [56].

### 2.3. Network Statistical Analysis

To analyze the core symptoms according to subjective cognitive decline, a network analysis was performed on 9 questions of the PHQ-9 for each group. The network represents a Gaussian graphical model [57], and all items of each measurement value are defined as ‘“nodes”. After controlling for all other items, the partial correlations between two items are marked as “edges”. In most cases, green and red edges symbolize positive and negative partial correlations, respectively. In this paper, a single color for positive partial correlations is presented. The wider and more saturated the edge is, the stronger the partial correlation. The thickest possible link corresponds to the maximum value of the strongest edge in the network and is displayed as the maximum value under the network. The closer the edge weight is to 0, the less saturated and smaller the edge [58].

After analyzing the network, between centrality, closeness centrality, strength centrality, and expected influence were calculated [59]. Intermediary centrality is a conceptualized value of the frequency at which a corresponding node appears on the shortest path between two nodes on a network. Proximity centrality is a value indicating the length of a path through which a specific node reaches other nodes. Mediation centrality is similar to proximity centrality, but if the latter purely measures the distance between nodes, the former differs in reflecting how many neighboring nodes a specific node has.

Intensity centrality is the sum of the absolute values of edge weights between a specific node and other nodes and indicates how closely the node is connected to other nodes in the network. Similar to intensity centrality, expected impact is similar in that it refers to how closely a node is connected to other nodes in the network; however, expected impact is negative because it is calculated by adding the relative values of edge weight association. Relationship metrics set a threshold in the range of network densities of 0.01 steps (Dmin: 0.01:0.50). The minimum density is that at which the two groups of networks are not fragmented and a path exists between each node and another node. The maximum intensity chosen is 0.50, since the plots are increasingly randomized thereafter [60]. In the network analysis conducted in this study, network metrics were calculated at each threshold value of (1) characteristic path length and (2) clustering coefficient. Clustering coefficients indicate how many possible connections are estimated across neighbor symptoms of a symptom of interest [61]. These coefficients also show how important the symptom is in connecting to and contributing to other networks.

In this study, the relationship between the SMC group, a cognitive discomfort group, and depression was analyzed through network analysis; in particular, the symptomatic characteristics of depression that were typical of the SMC group were identified. All analyses in this study were performed using JASP v0.16.2 (University of Amsterdam, Amsterdam, the Netherlands).

## 3. Results

### 3.1. General Characteristics

The average age of individuals in the SMC group was 73.75 years (SD = 8.19), which was significantly older than that of individuals without SMCs (70.88 years (SD = 7.78); *t* = 8.27, *p* < 0.001). For each group, there were 216 males (34.7%) and 406 females (65.3%) of SMC among the total SMC group and 1050 (36.6%) males and 1817 females (63.4%) in the no SMC group, indicating that the gender difference between the groups was not significant (X^2^ = 0.796, *p* = 0.382). Individuals in the SMC group showed significantly higher scores for all nine items of the PHQ-9 than those without SMCs (*p* < 0.001). The PHQ-9 item with the greatest difference between groups was Q6 (*t* = 16.526, *p* < 0.001, Table 1).

### 3.2. Network Analysis

#### 3.2.1. Interaction between Depressive Symptoms of SMCs

**Figure 1:**
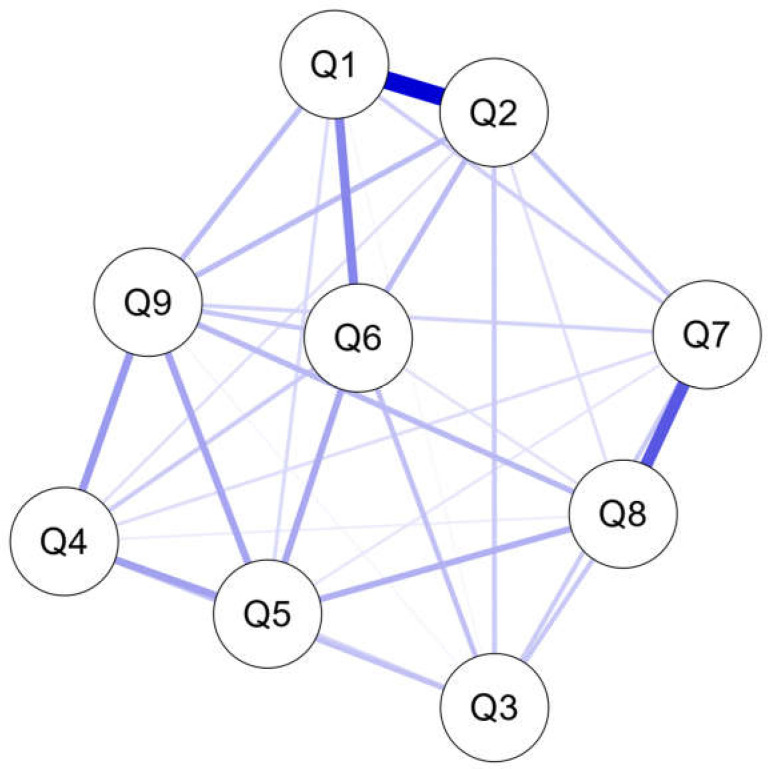
Network plots of depressive symptoms. Edge thickness indicated the strength of the partial correlation between nodes. Q1: anhedonia; Q2: depressed mood; Q3: sleep problems; Q4: low energy; Q5: ap-petite change; Q6: low self-esteem; Q7: concentration difficulties; Q8: psychomotor agitation or retardation; Q9: suicidal ideation.

Network analysis was performed on 9 nodes for depressive symptoms, and 33 edges out of 36 possible edges showed a significant association. Evaluating the connectivity between nodes first, the correlation between Q1 and Q2 in the SMC group was strongest, with r = 0.499, followed by that between Q7 and Q8 at r = 0.330 and between Q1 and Q6 at r = 0.239 (Figure 1 and Table A1 in Appendix A).

#### 3.2.2. Computing Centrality Measures of SMCs

**Figure 2:**
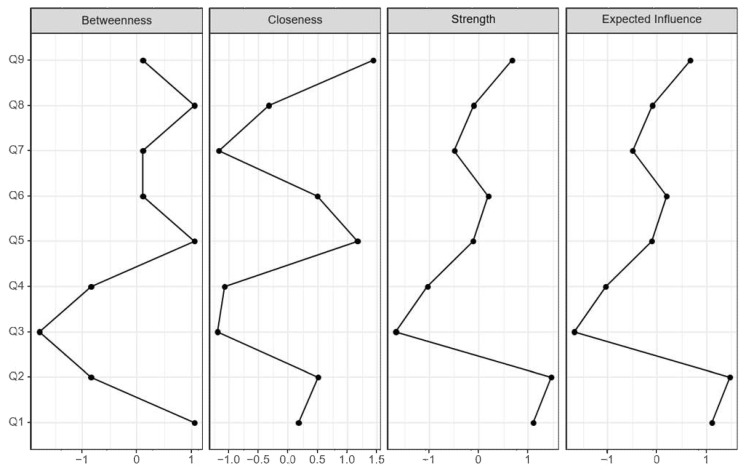
Centrality indices of the network of responses to depression in SMC. A high strength and expected influence values suggest that a node is positively associated with other nodes in the network (i.e., an increase in the value of that node is associated with an increase in the value of other nodes). Feeling down (PHQ-9 item 2) and having little interest or pleasure in doing things (PHQ-9 item 1) emerged as the most highly central symptoms.

The results of the standardized centrality analysis for each group are presented in Table 2 and Figure 2. The highest calculated centrality value was Q2 in strength and expected influence, followed by Q1 in all of betweenness, strength, and expected influence except for closeness. In the case of closeness, Q9 was the only high value, and the third equally high value was Q5, which showed a high level of betweenness and closeness (Figure 2 and Table 2).

#### 3.2.3. Clustering Coefficient Measures

**Figure 3:**
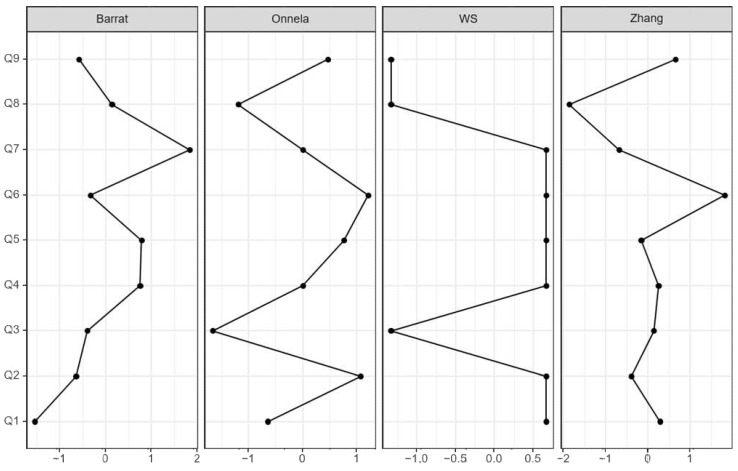
Clustering result.

The partial deciphering of structural and functional networks for depressive symptoms in both groups was measured by the tendency of nodes/factors to cluster together with Barrat [62], Onnela [63], Watts and Strogatz (WS) [61], and Zhang clustering coefficients [64]. The clustering coefficient indicates the strength of the connection between depressive symptoms and the subjective cognitive group. In the SMC group, the highest clustering coefficient values were Q7 (Barrat = 1.838), whereas the lowest was Q8 (Zhang = −1.869, Figure 3 and Table 3).

## 4. Discussion

In this study, we conducted a network analysis to determine how depressive symptoms appear in an elderly group with subjective discomfort. In addition, how the network pattern of depression is interconnected according to subjective cognitive decline was analyzed according to the most central and least central factors in the analyzed network pattern.

The network analysis indicated that the core depressive symptom was Q1, anhedonia, and all centrality measures showed a high value in common. Furthermore, Q2 was found to be another core symptom along with Q1. Q2 indicates feeling depressed, and Q1 and Q2 have a strong relationship in the network plot. People with SMCs may have emotion-based depressive symptoms; thus, they match the core symptoms of depressive disorder (depressed mood, anhedonia) of the DSM diagnostic criteria. The association shown in the network analysis was strong between concentration difficulties and psychomotor agitation or retardation (Q7 and Q8), between anhedonia and low self-esteem (Q1 and Q6), and between anhedonia and feeling depressed symptoms, but there was no high value in centrality.

The findings show that depressive symptoms act as indicators of subjective cognitive decline in old age through network analysis. Depression is usually associated with central serotonergic dysfunction and is centrally associated with the overall cognitive [65] and emotional experience of depression as it simply reflects the “feeling sad” aspect of depression [66]. Anhedonia as a symptom is lesser known than depressed mood, is associated with catecholaminergic dysfunction [65], and is sometimes accompanied by symptoms of MDD such as psychomotor agitation, excessive guilt or hopelessness, suicidal thoughts, and loss of weight or appetite. Anhedonia is also a key symptom of depression [67] and is observed in approximately 40% of patients with Parkinson’s disease [68,69]. Although anhedonia and depressed mood are both key symptoms of depression, they are empirically distinct in that they do not occur simultaneously [70], and there is a unique neural correlation [71].

In this study, both symptoms were found to be major depressive symptoms of SMCs as distinct endophenotypes of depression [65]. This suggests that the executive dysfunction and dysfunction of SMCs may be associated with persistent depression, perpetuating the self-reinforcing interaction between depression and cognitive decline [72,73]. In addition, in the case of depressed SMCs, attention and concentration ability, as well as frontal/executive function, were lower than those in the normal group [74,75]. As such, depression is a strong factor in SMCs and may be associated with greater neuropsychological damage to SMCs.

SMCs due to memory deterioration are thought to be difficult to objectively evaluate using subjective psychological reporting because depression, anxiety, and certain personality characteristics are related to education level [1,76,77,78]. Furthermore, in order for self-reporting on SMCs to be more clearly understood in the clinical field, it is necessary to answer questions such as “How accurate is an individual’s perception of his or her memory ability?” and “How valid is the questionnaire measuring subjective memory discomfort?” [79]. The SMCQ has high internal consistency and factor validity and shows that SMCs [80,81,82] are associated with objective cognitive function even after controlling for the effects of depression and demographic variables [75]. In addition, the frontal lobe/executive function in the SMC group was significantly lower than that in the normal group, even though it was in the normal category based on the objective cognitive test [75]. These results indicate that memory discomfort (e.g., AAMI) in old age is the same as objective memory performance, and even when SMCs are evaluated by subjective psychological reporting as evidence, those with SMCs appear to not worry much about memory deterioration.

The network analysis conducted in this study can identify highly “central” and influential symptoms, which are defined as having strong associations with other symptoms, through a “symptom-to-symptom” perspective [49,83,84]. Approximately 50% of patients with SMCs report a clinically significant level of depression [75], indicating that SMCs are related to emotional characteristics [85]. This finding was also found to indicate the relative importance of sad mood and anhedonia in depression in a recent network study, as these symptom centrality indices ranked highest among those of all depressive symptoms [86]. This analysis provides continuous and consistent results while being advanced, and the strength of the research is that it identifies the core symptoms among depressive symptoms appearing in SMCs.

The limitations of this study are as follows. First, the study involved community residents, and no clinical information about the subjects was available. Although symptoms of subjective cognitive decline were evaluated, there was no clinical information such as whether dementia was diagnosed. Next, there was no objective evaluation of cognitive function since a self-report test was utilized. In that the self-reported evaluation of cognitively impaired patients in a previous study was much more strongly related to the scores of personality items than performance on a neuropsychological test [87], the lack of a cognitive function evaluation result may indicate that a stronger relationship exists. Although the collected data were obtained from self-report tests, the limitations of these tests may have been overcome to some extent because the study was conducted by trained investigators, including mental health professionals and included a large sample.

Similar to the findings of previous studies, this paper found that depression was strongly associated with SMCs and identified the characteristic depressive symptoms of an SMC group. Confusion about the clinical manifestations of objective memory deficits in senile depression makes it difficult to distinguish different clinical manifestations and to determine treatment and prognosis [53]. Nevertheless, knowledge of the characteristic depressive symptoms associated with old age, which are strongly associated with memory loss, may be helpful for further screening and therapeutic intervention. In addition, unlike previous studies on depression and dementia, which are psychiatric symptoms that occur at a high rate in the elderly population, this study demonstrates the strength of being able to intervene by finding characteristic depressive variables targeting SMC, whose prevalence increases with age [88]. Therefore, this study differs in that SMC is implicitly a precursor to dementia and an intervention index for depression that can lead to suicide in the elderly.

## 5. Conclusions

Among the depressive symptoms in the SMC group, the most core symptoms were analyzed through network analysis. Depressed mood and anhedonia were found to be the strongest associations and key symptoms in elderly individuals with SMCs. In the future, interventions in elderly individuals with depressive symptoms can be expected to differ depending on whether or not SMCs are present.

## Figures and Tables

**Table 1:** Group differences in each PHQ-9 item.

Variable		No SMC (*n*= 2867)			SMC (*n* = 622)			SE Difference	Cohen’s d	*t*	*p*
		Mean	SD	SE	Mean	SD	SE				
Q1	Anhedonia	0.797	0.735	0.014	1.302	1.052	0.042	0.035	−0.631	−14.268	<0.001
Q2	Feeling depressed	0.81	0.767	0.014	1.357	1.102	0.044	0.037	−0.654	−14.777	<0.001
Q3	Sleep problems	1.028	0.98	0.018	1.608	1.25	0.05	0.046	−0.561	−12.69	<0.001
Q4	Low energy	0.786	0.763	0.014	1.278	1.121	0.045	0.037	−0.587	−13.28	<0.001
Q5	Appetite change	0.67	0.669	0.013	1.172	1.079	0.043	0.034	−0.662	−14.97	<0.001
Q6	Low self-esteem	1.004	0.887	0.017	1.698	1.194	0.048	0.042	−0.731	−16.526	<0.001
Q7	Concentration problems	0.727	0.666	0.012	1.185	1.02	0.041	0.033	−0.617	−13.949	<0.001
Q8	Agitation/Retardation	0.657	0.628	0.012	1.14	1.025	0.041	0.032	−0.675	−15.254	<0.001
Q9	Suicidal ideation	0.646	0.607	0.011	1.002	0.867	0.035	0.029	−0.539	−12.178	<0.001

**Table 2:** Centrality measures per variable in SMC.

Item	Betweenness	Closeness	Strength	Expected Influence
Q1	1.054	0.174	1.101	1.101
Q2	−0.843	0.504	1.464	1.464
Q3	−1.792	−1.195	−1.676	−1.676
Q4	−0.843	−1.075	−1.040	−1.040
Q5	1.054	1.169	−0.113	−0.113
Q6	0.105	0.489	0.189	0.189
Q7	0.105	−1.170	−0.496	−0.496
Q8	1.054	−0.328	−0.101	−0.101
Q9	0.105	1.432	0.671	0.671

**Table 3:** Clustering measures per variable in SMC.

Item	Barrat	Onnela	WS	Zhang
Q1	−1.550	−0.644	0.667	0.279
Q2	−0.645	1.071	0.667	−0.399
Q3	−0.400	−1.662	−1.333	0.137
Q4	0.751	−0.001	0.667	0.245
Q5	0.783	0.761	0.667	−0.163
Q6	−0.334	1.207	0.667	1.808
Q7	1.838	0.001	0.667	−0.689
Q8	0.137	−1.194	−1.333	−1.869
Q9	−0.579	0.462	−1.333	0.651

## Data Availability

The data presented in this study are available on request from the corresponding author. The data are not publicly available due to ethical restrictions.

## Appendix A

**Table A1:** Weights matrix in SMC.

Item	Q1	Q2	Q3	Q4	Q5	Q6	Q7	Q8	Q9
Q1	0								
Q2	0.499	0							
Q3	0.017	0.105	0						
Q4	0	0.062	0.121	0					
Q5	0.074	0	0.086	0.184	0				
Q6	0.239	0.134	0.123	0.101	0.17	0			
Q7	0.095	0.116	0.103	0.067	0.048	0	0		
Q8	0.006	0.063	0.108	0.037	0.156	0.06	0.33	0	
Q9	0.132	0.134	0.021	0.2	0.178	0.112	0.087	0.14	0
